# Expanding the VEXAS diagnostic workup: the role of peripheral blood cytological analysis

**DOI:** 10.3389/fimmu.2024.1466720

**Published:** 2024-10-03

**Authors:** Chiara Baggio, Francesca Oliviero, Roberto Padoan, Luca Iorio, Riccardo Bixio, Giovanni Orsolini, Eugenia Bertoldo, Cristina Bernardi, Davide Colavito, Barbara Paiero, Giovanna Pregnolato, Roberta Ramonda, Andrea Doria, Sara Bindoli, Paolo Sfriso

**Affiliations:** ^1^ Department of Medicine (DIMED), University of Padova, Padova, Italy; ^2^ Rheumatology Unit, University Hospital of Padova, Padova, Italy; ^3^ Rheumatology Unit, Department of Medicine, University of Verona, Verona, Italy; ^4^ Internal Medicine Unit, Department of Medicine, Mater Salutis Hospital, Legnago, Italy; ^5^ Rheumatology Unit, San Giovanni e Paolo Hospital, Venice, Italy; ^6^ R&I Genetics srl, Torre della Ricerca, Padova, Italy

**Keywords:** VEXAS, inflammation, hematology, cytology, cytokines, vacuoles

## Abstract

VEXAS syndrome is a newly described autoinflammatory entity characterized by somatic mutations in the UBA1 X-linked gene in hematopoietic progenitor cells. Several studies have demonstrated that the presence of vacuoles in progenitor cells from bone marrow aspirates is a hallmark finding for this syndrome. Therefore, this study aimed to characterize leukocytes from VEXAS patients versus patients with ANCA-associated vasculitis (AAV), familial Mediterranean fever (FMF), and healthy donors (HD) to define a specific cytological pattern that can support VEXAS diagnosis. Twelve VEXAS patients were included in the study. Blood samples from FMF (n = 16), AAV (n = 16) and HDs (n = 20) acted as controls. May-Grünwald Giemsa (MGG) staining was used for studying cellular morphology, including cytoplasm, granules, and vacuoles and to perform a cytogenic evaluation of leucocytes. Plasma IL-1β, IL-1α, TNFα, IL-18 and IL-8 were measured using ELISA assay. The cytological analysis from blood smears confirmed the presence of immature neutrophils in VEXAS patients. We found a greater number of vacuoles in VEXAS patients vs. FMF, AAV and HD. Micronuclei (MNi) and cell death rate were higher in VEXAS patients vs. HD. Cell death correlated with IL-1β and IL-8 levels. MNi were positively associated with IL-8 and IL-1β levels, and with the percentage of immature neutrophils and vacuoles. In conclusion, our findings suggested that cytological test may be supportive for VEXAS diagnosis, despite genetical analysis is mandatory for confirming the disease. Finally, we identified several cytological hallmarks that may distinguish the VEXAS “cytotype” not only from HD but also from other inflammatory diseases.

## 1 Introduction

VEXAS (vacuoles, E1 enzyme, X-linked, autoinflammatory, somatic) syndrome is an “hemato-autoinflammatory” disorder, associated with somatic mutations in the UBA1 gene (1). The syndrome is characterized by clinical features combining autoinflammatory features and myelodysplastic traits. The UBA1 gene mutations lead to E1 enzyme deficiency and impaired protein ubiquitylation, resulting in increased levels of unfolded protein, and thus elevated concentrations of proinflammatory cytokines (2). The most common pathogenic variants affect methionine at codon 41, although somatic mutations at splice sites may also affect UBA1 expression; among variants at codon 41, p.Met41Val is associated with a reduced UBA1b and increased UBA1c isoform (3), resulting in a worse phenotype. Although a definitive diagnosis of VEXAS syndrome requires genetic, this remains expensive and time-consuming. The evaluation of myeloid and erythroid progenitor morphology serves as a starting point for subsequent immunophenotypic, cytogenetic, and molecular investigations. Vacuoles found in bone marrow (BM) aspirates have been identified as a potential hallmark of VEXAS syndrome (2, 4–6), although vacuoles have been reported in other conditions (7–10). A few cases of VEXAS and atypical UBA1 mutations have been described with no or low levels of vacuoles in the BM precursors, suggesting a direct correlation between the residual activity of UBA1b and the presence or absence of vacuoles. Indeed, these patients had mild autoinflammatory manifestations, likely indicating that these mutations have a minimal effect on UBA1b function (2). Given the utility of BM aspirate analysis as a diagnostic tool for VEXAS syndrome, we supposed that cytological analysis of peripheral blood leukocytes could serve as a relevant starting point for guiding diagnosis, offering a simpler and less invasive screening method. In the present work, we characterized leukocytes in VEXAS versus patients with ANCA-associated vasculitis (AAV), familial Mediterranean fever (FMF), and healthy donors (HD). We also endeavored to investigate whether the leukocyte cytology in VEXAS patients is influenced by the nature of the UBA1 gene mutation.

## 2 Materials and methods

### 2.1 Patients included in the study

Twelve (100%) suspected VEXAS were included before genetic confirmation and tested in a cross-sectional manner. The median age was 75 years (IQR 72 - 81). Blood samples from 16 FMF, 16 AAV and 20 HDs acted as controls. Patients and comparison groups were not age- and sex-matched. Demographical data, clinical features, and genetics of VEXAS, FMF, AAV patients and HDs are reported in **Supplementary Table S1** and in **Supplementary Tables S3**-**S5**. HDs with no relevant comorbidities were chosen among healthcare workers of Padova University Hospital. We selected FMF and FMF-like patients (Eurofever criteria) (11), AAV patients (2022 ACR/EULAR criteria) (12) from the outpatient clinic of Padova University Hospital. All participants gave their written informed consent to participate in the study, which was conducted in compliance with the principles of the Declaration of Helsinki. The protocol follows the guidelines of the Ethics Committee of Padova University Hospital (protocol code 5349/AO/22).

### 2.2 Reagents

May-Grünwald and Giemsa staining, PBS, and methanol were from Sigma-Aldrich (St. Louis, Missouri). ELISA kits for IL-1β (sensitivity: 0.5 pg/mL), TNFα (sensitivity: 2 pg/mL) and IL-1α (sensitivity: 0.6 pg/mL) were from BioLegend (San Diego, California), total IL-18 (IL-18 and IL-18 bound to IL-18 binding protein, BP) (sensitivity: 11.7 pg/mL) was from R&D Systems (Minneapolis) and IL-8 (sensitivity: 2 pg/mL) was from Thermo Fisher Scientific (Waltham, Massachusetts).

### 2.3 Cellular morphology and cytogenic evaluation of leucocytes

Blood sample from VEXAS, FMF, AAV patients and HD was collected in EDTA tubes. May-Grünwald Giemsa (MGG) staining was used for studying cellular morphology and to perform a cytogenic evaluation of leucocytes (13). Oil immersion microscopy with 1000× magnification was applied for the cytological analysis. Leukocytes were identified based on morphology. For each smear, we examined at least 200 leucocytes and, in each field, the number of cell profiles corresponding to neutrophils (N), monocytes (M), lymphocytes (L), eosinophils and basophils were recorded. Results were finally expressed as a percentage of total leukocytes counted in a slide. Eosinophils’ morphology was evaluated using a consensus guideline explained in **Supplementary Table S2** (14). All slides were examined for the presence of vacuoles and different types of nuclear abnormalities (NA) including micronuclei (MNi) (15, 16), binucleated cells, hypersegmented N, karyolitic, karyorrhectic, and pyknotic cells (13). Finally, we also evaluated platelet size (small platelet, mean platelet diameter (MPD) < 2 µm; enlarged platelet MPD > 3.5 µm; giant platelet with MPD > 3.9 µm) and the presence of platelet clumps (17). A left shift indicates the presence of immature N in the blood. Immature N are usually band N, but also earlier forms can be seen (myelocytes, metamyelocytes). We calculated the Immature N/total N ratio (I/T) by dividing the percentage of immature N (bands, myelocytes and metamyelocytes) by the percentage of total N (both immature and mature). The study group was classified according to the I/T ratio into normal (< 0.2), moderate shift to the left (0.2 - 0.29) and severe shift to the left (≥ 0.3) (18).

### 2.4 Plasma collection and cytokine measurement

Blood from VEXAS, FMF and AAV patients and HD was collected in an EDTA tube and centrifuged for 5 minutes at 400g, finally, plasma was stored at -20°C. The following cytokines were measured in plasma using commercially available enzyme-linked immunosorbent assay (ELISA) kits: interleukin (IL)-1β, TNFα, IL-1α, total IL-18 and IL-8. The samples below the range of valid detection were reported in **Supplementary Table S6**, some differences could have gone undetected given the limited sensitivity of the ELISA assay kits.

### 2.5 Next-generation sequencing - whole exome sequencing

Whole blood from patients was collected for exome analysis after obtaining informed consent. The potential carriage of UBA1 mutations in HD, FMF and AAV patients was not assessed. DNA was extracted using the Qiagen BioRobot DNA extraction kit (Qiagen Benelux B.V., the Netherlands) according to the manufacturer’s instructions and quantified using Nanodrop spectral analysis (Thermo Fischer Scientific, Inc., Waltham, Massachusetts);. DNA fragmentation and degradation were evaluated by Tape Station technology (Agilent Technologies, Inc., Santa Clara, California). DNA Library preparation and whole exon enrichment were performed employing the Agilent All Exon V.6 kit (Agilent Technologies, Inc., Santa Clara, California). Library sequences were obtained using the HiSeq2500 Illumina Sequencer or Nextseq2000 Illumina Sequencer. Bioinformatics analysis included the following: NGS reads mapping to whole genomes using the Burrows-Wheeler Alignment tool with default parameters, polymerase chain reaction duplicate removal using Picard^®^, single nucleotide polymorphisms and indel calling using the Genome Analysis Toolkit (GATK) UnifiedGenotyper, variant annotation using snpEff and false positive variant filtration using the GATK VariantFiltration module. Exome sequencing data and reads alignment analysis were checked for coverage depth and alignment quality by employing the Bedtools software package. Variant analysis was performed employing bioinformatic prediction tools (Polyphen2, SIFT, MutationTaster, PhyloP, CADD-Phred);classification was conducted following the guidelines from the American College of Medical Genetics and Genomics.

### 2.6 Statistical analysis

The Shapiro-Wilk test was used to analyze the distribution of continuous variables, and variables with a non-normal distribution were presented as medians with the corresponding interquartile range (IQR). As no variable was normally distributed among the categories, only non-parametric tests were used. Kruskal-Wallis followed by Dunn’s *post hoc* tests were used for multiple comparisons. The differences between the two groups of patients were tested using the Mann-Whitney U test. Spearman correlation analysis was used to determine the correlations. Statistical analysis was performed with GraphPad Prism 8 (GraphPad Software Inc., La Jolla). A p-value < 0.05 was considered significant.

## 3 Results

### 3.1 Cellular morphology and cytogenic evaluation of leucocytes

**Figure 1:**
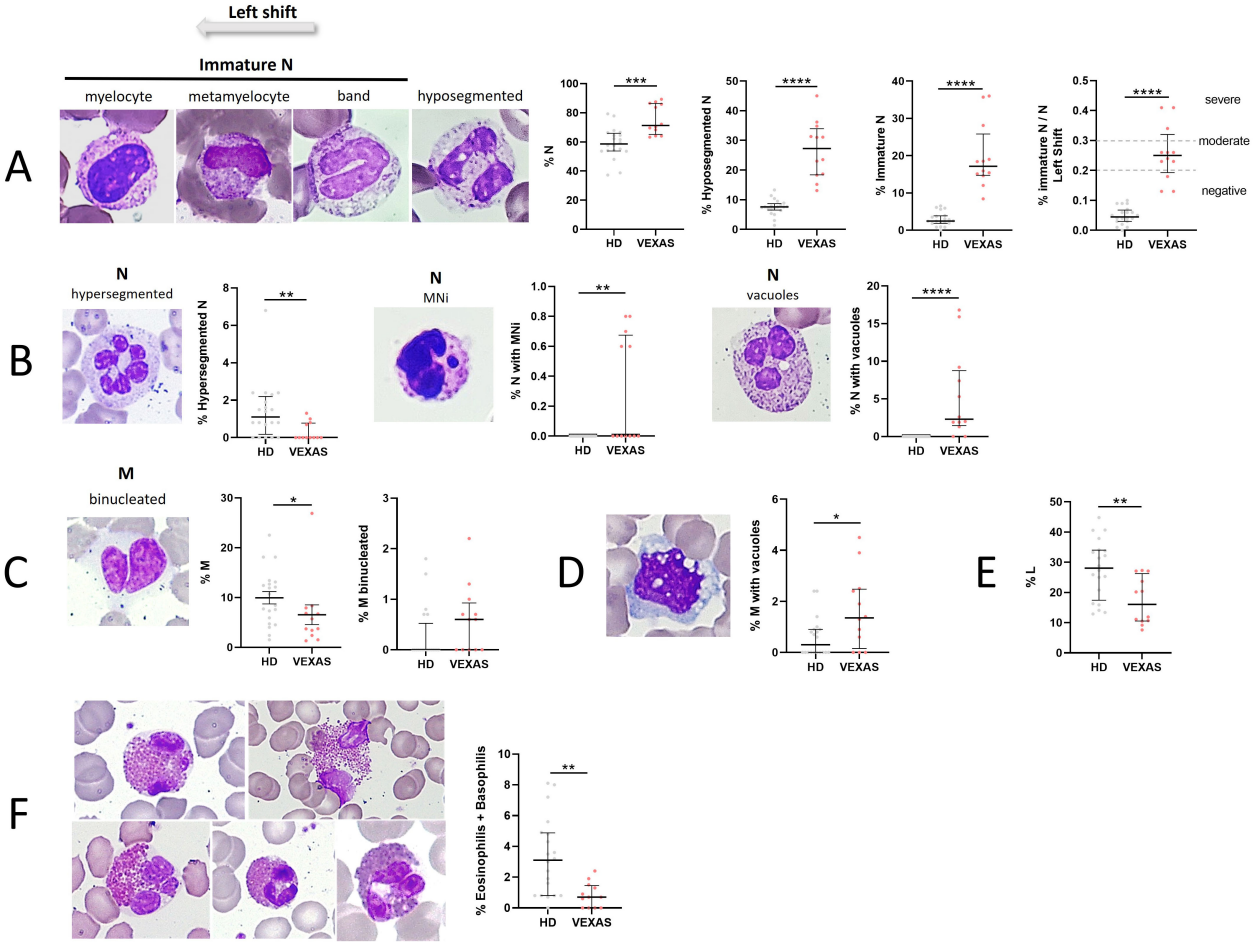
Cytogenic and cellular morphology evaluation of leukocytes in blood smear from VEXAS patients. Blood smears from VEXAS patients (n = 12) and HD (n = 20) were stained using MGG staining as described in Material and Methods. **(A)** Left panel: representative image of immature N and hyposegmented N stained with MGG. Right panel: rate of N, hyposegmented N and immature N in VEXAS patients and HD. **(B)** Left panel: representative image of hypersegmented N, N with MNi and vacuoles stained with MGG. Right panel: rate of hypersegmented N, N with MNi and vacuoles in VEXAS patients and HD. **(C)** Left panel: representative image of binucleated M stained with MGG. Right panel: rate of M and binucleated M in VEXAS patients and HD. **(D)** Left panel: representative image of M with vacuoles stained with MGG. Right panel: rate of M with vacuoles in VEXAS patients and HD. **(E)** Rate of L in VEXAS patients and HD. **(F)** Left panel: representative image of eosinophils stained with MGG. Right panel: rate of granulocytes (eosinophils and basophils) in VEXAS patients and HD. Data are expressed as medians and IQR. p calculated using the Mann-Whitney test: *p<0.05, **p<0.01, ****p<0.0001. MGG, May-Grünwald Giemsa; N, neutrophils; MNi, micronuclei; M, monocytes; L, lymphocytes; HD, healthy donors.

We evaluated the percentage of N, their stage of maturation and cytological characteristics in all blood smears from VEXAS patients. N were higher in VEXAS compared to those of HD (median values: 71.3 vs 58.6, p<0.0001). Circulating immature granulocytic precursors were found in all patients, in particular, hyposegmented N (neutrophils with pseudo-Pelger-Huët-like morphology) and immature N (band neutrophils, metamyelocytes and myelocytes) were higher in VEXAS compared to HD (median values: 17.2 vs 2.5, p<0.0001). A left shift indicates the presence of immature N in blood and usually indicates an inflammatory leukogram. 83% of VEXAS patients had abnormal I/T ratio (shift to the left). A moderate shift to the left (I/T ratio: 0.2 - 0.29) and severe shift to the left (I/T ≥ 0.3) were observed in 3 and 7 VEXAS patients, respectively and the I/T index was higher in VEXAS than in HD (median values: 0.3 vs 0.05, p<0.0001) (**Figure 1A**). Indeed, the hypersegmented N were lower in VEXAS compared to HD (median values: 0.0 vs 1.1, p<0.01) (**Figure 1B**). All the preparations were also examined for NA. N with MNi were observed only in 5 VEXAS patients, while they were absent in the N from HD (**Figure 1B**). Peripheral smears in most patients showed abnormal N with cytoplasmic vacuoles (n = 10), while vacuoles were absent in the N of HD (median values: 2.3 vs 0.0, p<0.0001) (**Figure 1B**). The percentage of M (median values: 4.7 vs 9.6, p<0.05), L (median values: 16.1 vs 28.1, p<0.01) and eosinophilic and basophilic granulocytes (median values: 0.7 vs 3.1, p<0.05) was lower in VEXAS compared to HD (**Figures 1C, E, F**). There were no significant differences in the rate of binucleated M between VEXAS and HD (**Figure 1C**) and the percentage of vacuolated M was higher in VEXAS compared to HD (median values: 4.5 vs 2.4, p<0.05) (**Figure 1D**). Although we observed fewer circulating eosinophils in VEXAS patients compared to HD, those observed showed alterations such as hyper lobated nucleus (code 2), agranular cytoplasm (code 5-6), vacuoles (code 7-8), and cell death (**Figure 1F**). The reduction in eosinophils and basophils observed in VEXAS smears is probably caused by the ongoing treatment primarily with glucocorticoids.

Among other mutations, the Met41Val variant seems to represent a risk factor for decreased survival in VEXAS and it has been found to cause a lower translation of UBA1b isoform when compared to the other mutations (1). Although non-significant, due to the low number of patients involved in this study, the analysis conducted by subdividing VEXAS according to different UBA1 mutations showed an increase in the percentage of N and immature N in patients with Met41Val mutation compared to VEXAS with other variants and HD. Furthermore, we did not detect hypersegmented N in VEXAS with the Met41Val mutation. Finally, the rate of N with vacuoles tended to be higher in VEXAS patients carrying Met41Val mutation than in VEXAS with Met41Thr, mutations at the splice acceptor site and HD. We found no differences between the different groups analyzed in the percentage of M with vacuoles, eosinophilic and basophilic granulocytes. Although non-significant, the percentage of L tended to be higher in patients with splice variant and Met41Thr compared to patients with Met41Val and Met41Leu mutations, and the percentage of M tended to be higher in patients with Met41Leu compared to the other VEXAS groups (**Supplementary Table S7**).

**Figure 2:**
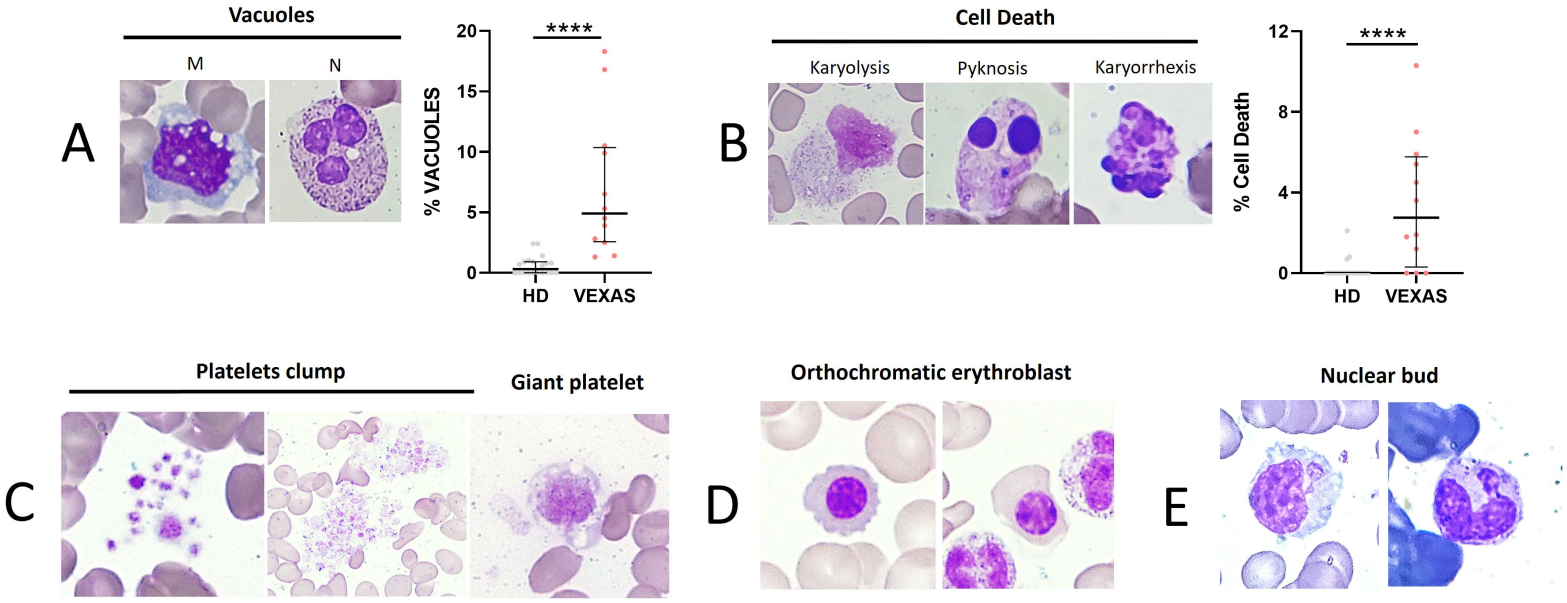
Cell death rate of leukocytes and vacuoles evaluation in blood smear from VEXAS patients. Blood smears from VEXAS patients (n = 12) and HD (n = 20) were stained using MGG staining as described in Materials and Methods. **(A)** Left panel: representative image of M and N with vacuoles stained with MGG. Right panel: rate of total vacuoles in VEXAS patients and HD. **(B)** Left panel: representative image of karyorrhexis, karyolysis, and pyknosis in blood smears stained with MGG. Right panel: rate of cell death in VEXAS patients and HD. **(C)** Platelet morphology and platelet clumps. **(D)** Orthochromatic erythroblast. **(E)** Nuclear bud. Data are expressed as medians and IQR. p calculated using the Mann-Whitney test: ****p<0.0001. MGG, May-Grünwald Giemsa; M, monocytes; N, neutrophils; HD, healthy donors.

As reported in the literature, VEXAS presented a higher number of total vacuoles in M and N compared to HDs (median values: 4.9 vs 0.3, p<0.0001) (**Figure 2A**). To monitor cytotoxic effects induced by inflammation, we evaluated the rate of cell death (karyorrhexis, karyolysis, and pyknosis) in leukocytes. A significant difference in the rate of cell death between VEXAS and HDs (median values: 2.8 vs 0.0, p<0.001) (**Figure 2B**) was found, as well as enlarged and giant platelets and platelet clumps in 6 VEXAS (**Figure 2C**). Orthochromatic erythroblast and nuclear bud were observed in 3 patients (**Figures 2D, E**).

Although non-significant, those carrying the Met41Val and Met41Leu variations had higher levels in the percentage of cells with vacuoles than those with Met41Thr and variants at the splice acceptor site. Finally, cell death levels tended to be higher in those with the Met41Val variant than in the other groups analyzed (**Supplementary Table S7**).

**Figure 3:**
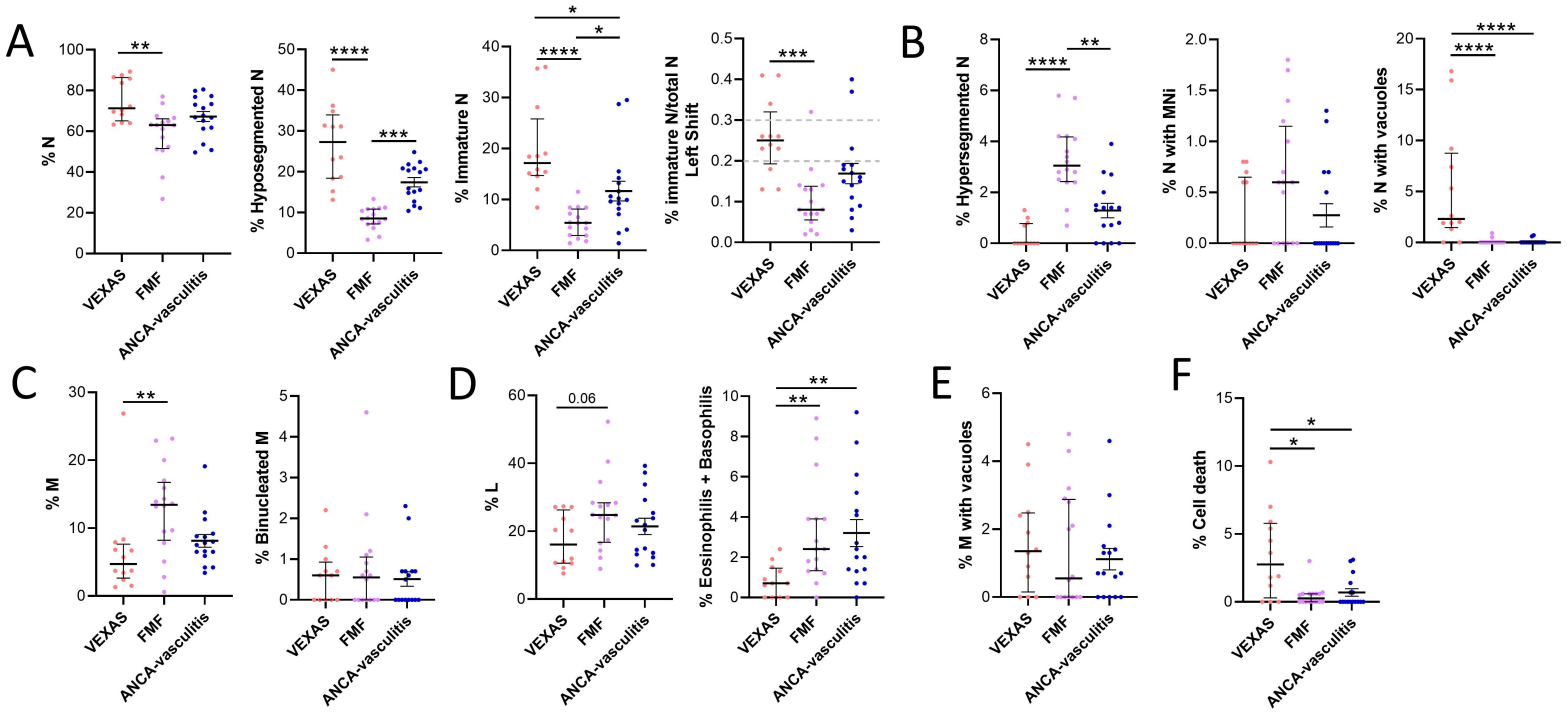
Cytogenic and cellular morphology evaluation of leukocytes in blood smear from VEXAS, FMF patients and patients with ANCA-associated vasculitis. Blood smears from VEXAS patients (n = 12), FMF (n = 16) and ANCA-associated vasculitis patients (n = 16) were stained using MGG staining as described in Materials and Methods. **(A)** rate of N, hyposegmented N and immature N in VEXAS, FMF patients ANCA-associated vasculitis patients. **(B)** Rate of hypersegmented N, N with MNi and vacuoles in VEXAS, FMF patients, ANCA-associated vasculitis patients. **(C)** Rate of M and binucleated M in VEXAS, FMF patients ANCA-associated vasculitis patients. **(D)** Rate of L and granulocytes (eosinophils and basophils) in VEXAS, FMF patients ANCA-associated vasculitis patients. **(E)** Rate of M with vacuoles in VEXAS, FMF patients, ANCA-associated vasculitis patients. **(F)** Rate of cell death in VEXAS, FMF patients, ANCA-associated vasculitis patients. Data are expressed as medians and IQR. p calculated using the Kruskal-Wallis test. Dunn’s *post hoc* test: *p<0.05, **p<0.01, ***p<0.001, ****p<0.0001. MGG, May-Grünwald Giemsa; N, neutrophils; MNi, micronuclei; M, monocytes; HD, healthy donors.

The cytology of VEXAS was compared affected by other inflammatory conditions such as FMF, FMF-like and AAV. N (median values: 71.3 vs 63.1, p<0.01), hyposegmented N (median values: 27.3 vs 8.5, p<0.0001) and immature N (median values: 17.2 vs 5.4, p<0.0001) were higher in VEXAS compared to FMF (**Figure 3A**). In addition, a higher shift to the left was observed in VEXAS compared to FMF (median values: 0.3 vs 0.1, p<0.001) (**Figure 3A**). Although non-significant, hyposegmented (median values: 27.3 vs 17.9) and immature N (median values: 17.2 vs 10.1) were also higher in VEXAS than in patients with AAV (**Figure 3A**). On the contrary, hypersegmented N was higher in FMF patients than in VEXAS (median values: 3.1 vs 0.0, p<0.0001). Although N with MNi were higher in FMF compared to VEXAS, these differences did not reach statistical significance. Unlike VEXAS (median value: 16.8), peripheral smears in most FMF (median value: 0.9) and AAV (median value: 0.7) did not show abnormal N with cytoplasmic vacuoles (p<0.0001) (**Figure 3B**). M were higher in FMF compared to VEXAS (median values: 13.5 vs 4.7, p<0.01) (**Figure 3C**). Of note, lower levels of granulocytes (eosinophils and basophils) were found in VEXAS compared to FMF (median values: 0.7 vs 2.4, p<0.01) and AAV (median values: 0.7 vs 2.2, p<0.05) (**Figure 3D**). Although not significant L (median values: 24.8 vs 16.1) was higher in FMF compared to VEXAS (**Figure 3D**). No differences were found in the levels of binucleated M and in monocytes with vacuoles (**Figures 3C–E**). Finally, the cell death rate was more expressed in VEXAS compared to FMF (median values: 10.3 vs 3.0, p<0.01) and AAV patients (median values: 10.3 vs 3.1, p<0.05) (**Figure 3F**).

### 3.2 Cytokine and chemokine evaluation in VEXAS patients and HD

**Figure 4:**
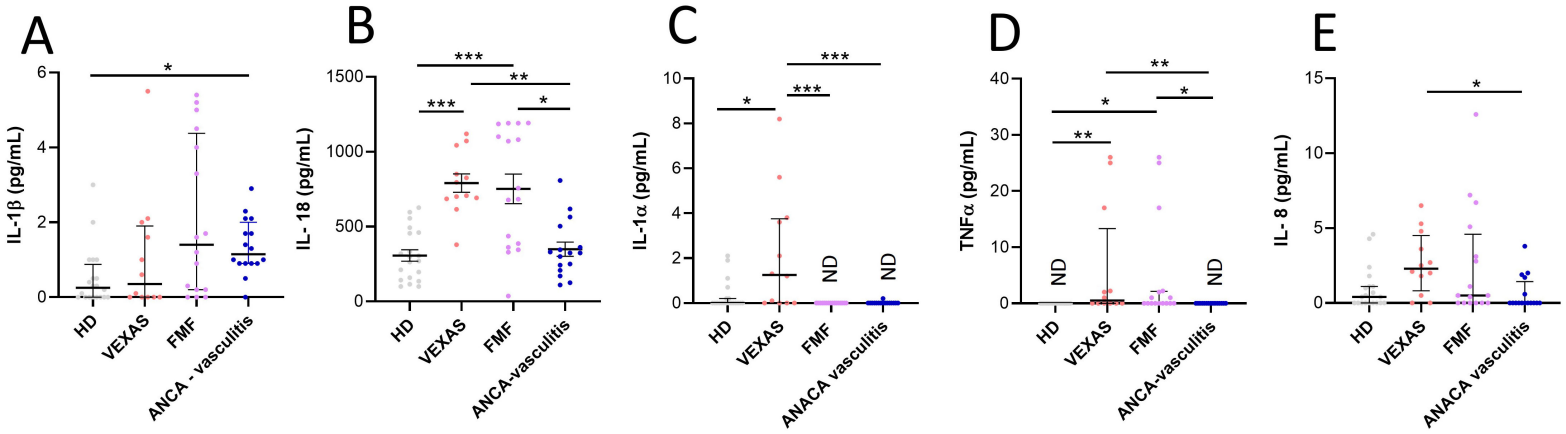
Cytokines and chemokines levels in plasma from VEXAS, FMF and AAV patients and correlation with cell death and I/T index. Cytokine and chemokines levels in plasma from VEXAS (n = 12), FMF (n = 16), AAV (n = 16) and HD (n = 20) were analyzed by ELISA as described in Materials and Methods. **(A)** IL-1β levels, **(B)** IL-18 levels, **(C)** IL-1α levels, **(D)** TNFα levels and **(E)** IL-8 levels. Data are expressed as medians and IQR. p calculated according using the Kruskal-Wallis test. Dunn’s *post hoc* test: *p<0.05, **p<0.01, ***p<0.001. HD, healthy donors.

No differences were found in the IL-1β levels between HD and VEXAS patients (**Figure 4A**). IL-18 (median values: 751 vs 285, p<0.0001), IL-1α (median values: 1.3 vs 0.0, p<0.05), TNFα (median values: 0.5 vs 0.0, p<0.01) and IL-8 (median values: 2.3 vs 0.4) levels were higher in VEXAS patients compared to HD, even if the significance is not reached for IL-8 levels (**Figures 4B–E**). Furthermore, IL-1α levels were higher in VEXAS (median values: 1.3) than in FMF (median values: ND) and AAV (median values: ND) patients and, IL-18, TNFα and IL-8 levels were higher in VEXAS (median values IL-18: 751, TNFα: 0.5, IL-8: 2.3) than in AAV (median values IL-18: 326.6, TNFα: ND, IL-8: 0.0) patients (**Figure 4**). Cytokine levels subdivided according to the mutations observed are shown in **Supplementary Table S8**. Although non-significant, due to the small sample size, VEXAS with the Met41Val variation had higher IL-1β, IL-18, IL-1α and IL-8 levels than those carrying the Met41Thr, Met41Leu or acceptor site splice mutations. The highest TNFα levels were found in 2 patients carrying the Met41Val, and in one patient with the Met41Thr mutation (26 pg/mL). The I/T index was positively correlated with IL-18 (r = 0.697, p = 0.014), IL-1α (r = 0.566, p = 0.059) and TNFα levels (r = 0.659, p = 0.023). The percentage of cell death was positively correlated with IL-1β (r = 0.840, p = 0.001) and IL-8 levels (r = 0.758, p = 0.006). Finally, IL-8 levels positively correlated with the vacuoles (r = 0.732, p = 0.009) and MNi rate (r = 0.777, p = 0.005). Also, IL-1β levels were positively associated with the MNi rate (r = 0.548, p = 0.071), although this correlation is not significant for IL-1β due to the low number of patients involved in the study. Conversely, In FMF and AAV patients IL-1β levels did not correlate with cell death (FMF: r = 0.043, p = 0.872; AAV: r = 0.243, p = 0.362) and MNi rate (FMF: r= 0.0004, p = 0.989; AAV: r = 0.0005, p = 0.985)”. In FMF and AAV patients also IL-8 levels did not correlate with cell death (FMF: r = 0.190, p = 0.473; AAV: r = - 0.220, p = 0.434), MNi rate (FMF: r = - 0.380, p = 0.146; AAV: r = 0.165, p = 0.560) and vacuoles (FMF: r = 0.391, p = 0.134; AAV: r = - 0.223, p = 0.404). In addition, the MNi rate positively correlates with the percentage of N (r = 0.599, p = 0.044*) and immature N (r = 0.591, p = 0.047*) and with the rate of vacuoles (r = 0.705, p = 0.014*).

## 4 Discussion

The characteristic peripheral blood leukocytes features in VEXAS can be a useful tool to distinguish this disease from other inflammatory diseases while awaiting for genomic test results. Cytological analysis has the advantage of being a rapid and easily accessible analysis to use in a clinically suspected patient. Our study aimed to compare leukocytes from VEXAS patients versus leukocytes from patients with AAV and FMF, as well as HD to define a specific cytological pattern which may facilitate and the diagnosis in patients with clinical manifestations suggestive of VEXAS syndrome.

Generally, blood abnormalities are frequently seen in VEXAS; VEXAS indeed is strictly related to Myelodysplastic Syndrome (MDS) and often the two diseases overlap. The incidence of VEXAS associated-MDS is estimated between 25-55% of the cases, with a prevalence of low-risk MDS (19). Furthermore, in a recent large representative diagnostic cohort of MDS patients, 1% of patients were found to be carriers of likely pathogenic *UBA1* mutations, underlying the importance to consider *UBA1* mutations in the diagnostic workup for MDS and MDS/VEXAS overlap (20). Our entire cohort had haematological abnormalities, primarily represented by macrocytic anemia, while five patients (42%) had low-risk MDS, confirmed by bone marrow biopsy (BMB). The leakage of marrow precursors into the bloodstream is a common occurrence in high-grade inflammatory states. Circulating immature granulocytic precursors were found in all patients of our cohort.

VEXAS disease course is characterized by progressive cytopenia with an associated risk of developing malignancies such as acute myeloid leukaemia (6, 21), though the underlying pathogenetic mechanisms are still unknown. One of the best-validated biomarkers of genomic instability is the presence of MNi. Our data showed an increase in NA (i.e. cells with MNi and buds) in VEXAS vs. HD. We found a positive correlation between the rate of MNi and the rates of N and immature N as well as with inflammatory cytokines IL-1β and IL-8. Interestingly, cells with MNi were also associated with the percentage of vacuoles, suggesting that excessive chromosome instability may stem from oxidative DNA damage. Various studies have shown the correlation between MNi and inflammation. It has been reported that inflammation can induce MNi which in turn can induce inflammation via the cGAS-STING cascade, leading to the release of interferon type I cytokines and the activation of NLRP3 inflammasome (13, 22). Based on our data, we hypothesized that in VEXAS, chronic inflammation may induce chromosomal instability which, in turn, may be responsible for the progression to hematological malignancy. In addition, MNi may directly contribute to the enhancement of inflammation by promoting the release of pro-inflammatory cytokines (23). Another recent and rather promising hypothesis is that defects in the UBA1 gene which is largely involved in the cellular DNA damage response machinery might induce chromosomal instability and facilitate the progression towards a hematological condition (21, 24).

All the twelve patients displayed an increase in the mean corpuscular volume (MCV) with a concurrent anemia. Chronic inflammation may be responsible for anemia, but the exact mechanism remains unclear (21). We found erythroid precursors (orthochromatic erythroblasts) only in 3 VEXAS and not in HD, FMF or AAV patients. One plausible explanation is that the erythroblasts found in the peripheral blood of these patients are due to mutations in UBA1 that can be involved in the maturation process of red blood cells. During the erythropoiesis, erythroid precursors activate a program of degradation that involves ubiquitination, to eliminate unnecessary proteins and their nucleus (21, 25).

Other forms of cytopenia (e.g. lympho/monocytopenia) might also be observed and may be responsible for the increased risk of infections (21). We found lower percentages of M and L in VEXAS vs. HD, FMF and AAV patients (6). The risk of infection has a double component, related to both the disease characteristics and the employment of high-dose steroids and immunosuppressants such as methotrexate or JAK-inhibitors. One patient treated with the Janus kinase (JAK) inhibitor filgotinib, developed osteomyelitis from *L. Monocytogenes* and *P.mirabilis* requiring multiple antibacterial treatments. Two other patients had severe pulmonary infections, whereas a third patient had bacterial endocarditis. Moreover, SARS-CoV-2 infection was reported in six (50%) patients, albeit only one of them developed severe complications.

The first description of VEXAS syndrome reported distinctive vacuolization in neutrophil and erythroid precursors. However, isolated vacuolization in BM smears might be observed in various contexts (21, 25, 26). Recently, it has been reported that a threshold of 10% of vacuoles in neutrophilic precursors provided high sensitivity and specificity for identifying VEXAS syndrome (2, 27–29),while the quantitative assessment of the proportion of vacuolized precursors on bone marrow smears help to identify the disease (27). Although BM aspirate remains a pivotal procedure, we highlighted how the cytological analysis from peripheral blood is a less invasive method and could constitute a relevant starting point to guide towards the proper diagnosis. An increased number of vacuoles was detected in N and M from VEXAS patients vs. HD. Notably, vacuoles in N were found only in VEXAS and not in HD, being even higher than in FMF and AAV, suggesting that the presence of vacuoles in precursors may identify primarily with these patients compared to other inflammatory known conditions. Future studies will be done on active FMF and AAV patients to exclude that neutrophil vacuolation is a nonspecific sign of inflammation. However, in 2 patients carrying the Met41Thr and c.118-2A>G variants respectively, we did not detect vacuolization. A few cases of individuals with VEXAS syndrome without vacuoles in the BM precursors, which might be linked to a specific UBA1 mutation, have been described. Therefore, the absence of vacuoles in BM precursors in VEXAS syndrome is possible and should not exclude the diagnosis of VEXAS (2). The absence of vacuoles in the peripheral smear does not exclude the diagnosis of VEXAS, and, furthermore, does not indicate the absence of vacuoles in the BM precursors.

A limitation of the study regards the comparison groups that were not age- and sex-matched. Although, Groarke EM et al. reported that many changes occur in the bone marrow with increasing age (alterations in overall cells number, decrease in lymphoid progenitors, senescence, lineage differentiation, cellular composition, and function of the hematopoietic stem cell) and these variations may be reflected in a different white blood cell count; no study highlighted cytological differences in the rate of vacuoles, nuclear abnormalities and abnormal neutrophilic subset related to the age (26).

Ubiquitination is critical for innate immune responses as it regulates inflammatory pathways, particularly the NF-κB pathway, immune homeostasis, survival, and DNA repair (21). Kosmider et al. reported the presence of activated inflammatory pathways and a significant increase in circulating levels of IL-1β and IL-18 in monocytes of VEXAS vs. controls, including inflammatory disorders without UBA1 mutations (29). Furthermore, Wu et al. reported that mtUBA1 myeloid cells upregulate inflammatory pathways compared with wild-type cells (30).

Higher levels of proinflammatory cytokines (IL-18, IL-1α, TNFα and IL-8) were observed in VEXAS than in HD, but no differences in IL-1β levels of HDs and VEXAS were detected. IL-18, IL-1α and TNFα levels were positively associated with the I/T index suggesting that immature neutrophils may impair innate immune functions. IL-18 supports neutrophil accumulation (influx of immature neutrophilis) and activation, promoting early innate immune responses and the amplification of immune response (31). In this work, total IL-18 levels were reported (IL-18 and IL-18 bound to IL-18 binding protein, BP). Therefore, considerations made on this cytokine must take into account that the IL-18 levels reported also include the not biologically active cytokine (IL-18 bound to IL-18BP).

The ubiquitin system also regulates programmed cell death mechanisms (32), and increased in apoptosis, pyroptosis, and necroptosis signatures in monocytes from VEXAS patients vs. other groups were reported, reflecting the inflammasome activation (29). Similarly, in our cohort, we found a significant difference in the rate of cell death in VEXAS, and the percentage of karyolysis, karyorrhexis and pyknosis correlated with IL-1β and IL-8 levels. Notably, the cell death rate was higher in VEXAS patients vs. FMF and AAV.

Promising data suggests that the phenotype of VEXAS syndrome differs according to the nature of the mutation, maybe due to a different translation rate of the UBA1b isoform starting from codon 41 localized in the cytoplasm (21, 27). Based on this, we set out to ascertain whether the leukocyte cytology may be influenced by the nature of the mutation. Our analyses revealed differences between patients with different mutations, particularly the Met41Val mutation, which may predispose to a more severe disease phenotype. Unfortunately, we were not able to perform a statistical analysis due to the small sample size. According to the current literature, Met41Val appears to cause a greater reduction in residual UBA1b translation as compared to Met41Leu or Met41Thr, and this genotype has been associated with a worse clinical outcome than other variants (32, 33). Our patients with Met41Val mutation exhibited no clinical or biological differences vs. other variants, although the sample size is too small to draw any satisfactory conclusions. Overall, the percentage of N and immature N in patients with Met41Val mutation was higher compared to patients with other variants; and the rate of N with vacuoles and cell death tended to be higher in those carrying the Met41Val mutation vs. Met41Thr or splice mutations. Finally, higher levels of proinflammatory cytokines and chemokines (IL-1β, IL-18, IL-1α, TNFα and IL-8) were found in Met41Val patients.

Our findings suggest that cytological test may be supportive for VEXAS diagnosis, despite genetical analysis is mandatory for confirming the disease. That is an important point, particularly given that patients may be at risk of rapid worsening and disease progression. Several cytological hallmarks may distinguish the VEXAS “cytotype” from healthy subjects, but also from other inflammatory conditions. This study has some limitations due to the small sample size and the diagnostic delay, which prevented patients from not being treatment-naive during the cytological evaluation. Cytological abnormalities found in AAV and FMF patients may be different in patients with active disease and, further studies, will allow us to evaluate these aspects. Further investigations in larger cohorts are needed to stratify patients, in particular for predictive purposes and response to therapy.

## Data Availability

The raw data supporting the conclusions of this article will be made available by the authors, without undue reservation.
