# Supplementary material for: Expanding the VEXAS diagnostic workup: the role of peripheral blood cytological analysis
Source: Front Immunol. 2024 Oct 3;15:1466720. doi: 10.3389/fimmu.2024.1466720 (PMC11484077; doi:10.3389/fimmu.2024.1466720)
Supplement: Supplementary file 1 [file Table1.docx]

Supplementary Material

**Supplementary Table S1.** Clinical, genetical and laboratory characteristics of the twelve VEXAS male patients who underwent peripheral cytology evaluation.

| **Age** (years), **sex** | | **UBA-1 mutation** | **Clinical Features** | **Laboratory exams** | **Infections occurred** | **Hematological features** | **Therapy** | **Relevant comorbidities** |
| --- | --- | --- | --- | --- | --- | --- | --- | --- |
| 66  M | p.Met41Leu  (c.121 A>C) | | fever  polychondritis  tenosynovitis  right iliac-femoral DVT  orchitis  leukocytoclastic vasculitis (lower limbs purpura)  neutrophilic dermatosis  pleural effusion | Macrocytic anemia  Increase in CRP and ferritin | SARS-CoV-2 (moderate) | Low-risk MDS  MGUS | Methyl-prednisolone  16 mg/day | No |
| 81  M | p.Met41Leu  (c.121 A>C) | | fever  urticarial rash  auricular chondritis  lower limbs sensorimotor neuropathy and upper limbs axonal sensory neuropathy  arthritis (hands and feet)  pulmonary thickening and subsequent pleural effusion  ocular inflammation | Macrocytic anemia  Increase in ferritin | SARS-CoV-2 (moderate) | Low-risk MDS | Prednisone  10 mg/day | Aorto-right femoral and left hypogastric bypass (Leriche syndrome) |
| 85  M | c.118-1G>C  p.? | | fever  arthralgias  pulmonary NSIP  multiple DVT (lower and upper limbs)  auricular chondritis | Macrocytic anemia  Increase in CRP and ferritin Increase in SAA | Hip osteomyelitis from Listeria Monocytogenes and Proteus Mirabilis  SARS-CoV-2 (mild) | BMB not performed | Prednisone  15 mg/day  Methotrexate  10 mg/week  Filgotinib 200mg/day | CPPD arthritis |
| 80  M | p.Met41Val  (c.121 A>G) | | fever  orchitis  unspecific lower limb rash  arthralgias  periorbital edema  bilateral lower limb DVT | Macrocytic anemia  Increase in CRP at disease onset | Bacterial endocarditis  Urosepsis | BMB not performed | Prednisone  10 mg/day  Methotrexate  15 mg/week | Pancreatic IPMN |
| 71  M | p.Met41Thr  (c.122 C>T) | | fever  recurrent lower limb purpuric lesions  neutrophilic dermatosis  arthralgias  asthenia/weight loss  nasal chondritis  mild pleural effusion  left leg SVT | Macrocytic anemia  Increase in CRP and ferritin  Increase in SAA  Thrombocytopenia | SARS-CoV-2 (mild) | Low-risk MDS | Prednisone  10 -15 mg/day | Arterial hypertension  Hyperuricemia  Pulmonary arterial hypertension  Parapsoriasis and lichenoid pityriasis |
| 68  M | c.118-2A>G | | polychondritis  sensorimotor polyneuropathy  lower limbs DVT  vasculitic-like lesions at lower limbs  serositis | Macrocytic anemia  Increase in CRP  Increase in SAA | No | BMB not performed | Prednisone  10 mg/day,  Methotrexate  20 mg/week | anti MAG-2 antibodies |
| 74  M | p.Met41Thr  (c.122 T>C) | | erythema nodosum  aortitis  episcleritis  nasal, auricular and possibly trachea polychondritis | Macrocytic anemia | Legionella pneumophila  (cause of death) | No MDS | Prednisone  12.5 mg/day | Cardiac ablation for PSVT |
| 85  M | p.Met41Thr  (c.122 T>C) | | fever  relapsing polychondritis  skin vasculitic lesions  erythema nodosum-like lesions | Increase in CRP | No | No MDS | Prednisone  25 mg/day | Arterial hypertension  Basal cell carcinoma |
| 75  M | p.Met41Val  (c.121 A>G) | | fever  erythema nodosum  thrombophlebitis episodes | Macrocytic anemia  Increase in CRP | No | MDS | Prednisone  18.75 mg/day | No |
| 73  M | p.Met41Thr  (c.122 T>C) | | fever  arthritis  low back pain  lower limbs purpura  left saphenous SVT  conjunctival hyperemia | Macrocytic anemia  Increase in CRP | SARS-CoV-2 (mild) | Low-risk MDS | Prednisone  25 mg/day | Type 2 Diabetes  NSTEMI treated with PTCA  severe hypertriglyceridemia |
| 77  M | p.Met41Thr  (c.122 T>C) | | sensorimotor polyneuropathy  Sweet syndrome  lower limbs DVT  episcleritis  sensorineural hearing loss | Macrocytic anemia  Increase in CRP and ferritin at disease onset  Thrombocytopenia | SARS-CoV-2  (severe with IMV) | Low-intermediate risk MDS  MGUS | Methyl-prednisolone  8 mg/day | Hemicolectomy for diverticulitis |
| 73  M | p.Met41Thr  (c.122 T>C) | | asthenia/weight loss  auricular and nasal chondritis  episcleritis | Macrocytic anemia  Increase in ferritin | Pneumonia from Herbaspirillum huttiense | No MDS | Methyl-prednisolone 24 mg/day  canakinumab  150 mg/4 weeks | NSTEMI treated with PTCA  Previous vertebroplasty  Atrial fibrillation (ablated)  Epilepsy  Panlobular emphysema  Lung neoformation in the right upper lobe  Hypothyroidism secondary to amiodarone |

Abbreviations are as follows: BMB, bone marrow biopsy; CRP, C-reactive protein; CPPD, calcium pyrophosphate crystal deposition; DVT, deep vein thrombosis; IPMN, intraductal papillary mucinous neoplasm; IMV, invasive mechanical ventilation; Hb, haemoglobin; MCV, mean corpuscle volume; MDS, myelodysplastic syndrome; MGUS, monoclonal gammopathy of undetermined significance; NSIP, nonspecific interstitial pneumonia; NSTEMI, non-ST-elevation myocardial infarction; PSTV, Paroxysmal supraventricular tachycardia; PTCA, percutaneous transluminal coronary angioplasty; SAA, serum-amyloid A; SARS-CoV-2, severe acute respiratory syndrome Coronavirus 2; SVT, superficial vein thrombosis.

**Supplementary Table S2**. Consensus guideline for evaluation of eosinophil morphology [14].

| **Code** | **Description of eosinophil morphology** |
| --- | --- |
| **0** | Normal: the nucleus has two or three lobes, the cytoplasm is not degranulated and there are few if any vacuoles. |
| **1** | Nonlobated nucleus: mature eosinophil with no lobation, the nucleus in the form of a band (a) or nucleus round/oval (b). |
| **2** | Hyperlobated nucleus: four or more nuclear lobes. |
| **3** | Ring nucleus. |
| **4** | Binuclearity. |
| **5** | Moderately hypogranular but agranular cytoplasm is less than 25% of the cytoplasmic area. |
| **6** | Markedly hypogranular: agranular cytoplasm is 25% or more of the cytoplasmic area. |
| **7** | Moderately vacuolated but vacuoles occupy less than 25% of cytoplasm. |
| **8** | Markedly vacuolated: vacuoles occupy 25% or more of cytoplasm. |
| **9** | Some granules with basophilic staining characteristics. |

**Supplementary Table S3.** Clinical and laboratory characteristics of the FMF and FMF-like patients who underwent peripheral cytology evaluation.

| **Characteristics of patients** | n = 16 |
| --- | --- |
| **Sex, n %**  Male  Female | 5 (31.3)  11 (68.7) |
| **Age at sampling, y, median [IQR]** | 33 [24.5 - 40.3] |
| **MEFV gene mutation, n %**  M680I, E148Q hz  K695R hz (3)  R202Q hz (4)  R202Q homo (2)  E148Q hz (1)  E148Q, P369S hz (1)  M694I, V726A hz (1)  M680I homo (1)  E195D hz (1)  **MVK, IFIH1, TNFRSF1A gene mutation, n %**  MVK (1)  IFIH1 (1)  TNFRSF1A (1) | 2 (12.5)  3 (18.7)  4 (25.0)  2(12.5)  1 (6.3)  1 (6.3)  1 (6.3)  1 (6.3)  1 (6.3)  1 (6.3)  1 (6.3)  1 (6.3) |
| **Disease activity, n %**  Relapsing-remitting  Remission | 8 (50)  8 (50) |
| **Therapy, n %**  Colchicine 1-2 mg/day  Anakinra 100 mg/day  Canakinumab 150-300 mg/4 weeks  None | 7 (43.7)  2 (12.5)  4 (25.0)  3 (18.8) |

Abbreviations are as follows: FMF, Familial Mediterranean Fever; IQR, interquartile range; y, years; n, number.

**Supplementary Table S4**. Characteristics of the AAV patients who underwent peripheral cytology evaluation.

| **Characteristics of patients** | n = 16 |
| --- | --- |
| **Sex, n %**  Female  Male | 6 (37.5)  10 (62.5) |
| **Age at sampling, y, median [IQR]**  **ANCA positivity at diagnosis, n %**  **PR3 positivity, n %**  **MPO positivity, n%** | 55 [42 - 72]  16 (100)  8 (50)  10 (62.5) |
| **Diagnosis, n%**  GPA  MPA | 8 (50)  8 (50) |
| **Disease duration at sampling, m, median [IQR]**  **BVAS v3 at sampling, median [IQR]**  **VDI at sampling, median [IQR]** | 61 [42.5 - 122]  0 [0 - 0]  4 [2 - 4.8] |
| **Time from OCS discontinuation, m, median [IQR]** | 33 [6.5 - 53] |
| **Patient on IST at sampling, n, %**  **IST duration at sampling, m, median [IQR]** | 14 (87.5)  24 [3 - 42] |

Abbreviations are as follows: AAV, ANCA associated vasculitis; ANCA, Anti-neutrophil cytoplasmic antibodies; BVASv3, Birmingham vasculitis score version 3; GPA, granulomatosis with polyangiitis; IQR, interquartile range; IST, immunosuppressive therapy; m, month; MPA, micropolyangiitis; MPO, myeloperoxidase; n, number; OCS, oral cortico-steroids; PR3, proteinase 3; VDI, vasculitis disease index; y, years.

**Supplementary Table S5.** Characteristics of HDs who underwent peripheral cytology evaluation.

| **Characteristics of patients** | n = 20 |
| --- | --- |
| **Sex, n %**  Female  Male | 10 (50%)  10 (50%) |
| **Age at sampling, y median [IQR]** | 33 [29 - 58] |

Abbreviations are as follows: IQR, interquartile range; y, years.

**Supplementary Table S6.** samples below the range of valid detection.

|  | **HD** | **VEXAS** | **FMF** | **AAV** |
| --- | --- | --- | --- | --- |
| **IL-1β** | 13 | 6 | 6 | 1 |
| **IL-18** | 0 | 0 | 0 | 0 |
| **IL-1α** | 16 | 5 | 16 | 16 |
| **TNFα** | 20 | 7 | 11 | 16 |
| **IL-8** | 18 | 3 | 10 | 12 |

**Supplementary Table S7.** Cytogenic and cellular morphology evaluation of leukocytes in blood smear from VEXAS patients differentiated according to UBA1 mutations.

|  | **HD** | **Met41Leu** | **Met41Thr** | **Met41Val** | **splice mutations** | **p** |
| --- | --- | --- | --- | --- | --- | --- |
| **N, % (IQR)** | 59 (54 - 66) | 75 (64 -86) | 69 (64 - 85) | 87 (87 - 88) | 71(70 - 72) | 0.008 |
| **Hyposegmented N, % (IQR)** | 7.6 (6.5 - 8.7) | 26 (15 -36) | 27 (22 - 35)** | 25(18 - 31) | 24 (13 - 35) | 0.0002 |
| **Immature N, % (IQR)** | 2.5 (1.8 - 3.9) | 15 (15-16) | 16 (11 - 21)** | 36 (36) | 19 (18 - 19) | 0.0001 |
| **Hypersegmented N, % (IQR)** | 1.1 (0.2 - 2.2) | 0.5 (0.0 - 1.0) | 0.0 (0.0 - 0.7) | 0.0 (0.0) | 0.7 (0.0 - 1.3) | 0.0849 |
| **N with vacuoles, % (IQR)** | 0.0 (0.0) | 9.4 (2.0 - 17) | 2.3 (1.4 - 5.8)** | 13 (9.2 - 16) | 0.7 (0.0 - 1.3) | <0.0001 |
| **M, % (IQR)** | 9.6 (5.0 - 13) | 15 (3.4 - 27) | 5.3 (3.2 - 8.0) | 1.9 (1.3 - 2.4) | 6.2 (5.7 - 6.7) | 0.0915 |
| **M with vacuoles, % (IQR)** | 0.3 (0.0 - 0.9) | 2.3 (0.0 - 4.5) | 1.2 (0.5 - 2.1) | 1.9 (1.3 - 2.4) | 2.0 (0.0 - 3.9) | 0.2797 |
| **L, % (IQR)** | 28 (17 - 34) | 9.2 (7.6 - 11) | 24 (11 - 27) | 11 (10 - 11) | 22 (20 - 24) | 0.0165 |
| **Granulocyte, % (IQR)** | 3.1 (0.8 - 4.9) | 0.8 (0.0 - 1.5) | 0.8 (0.5 - 1.6) | 0.4 (0.0 - 0.7) | 1.0 (0.0 - 1.9) | 0.0354 |
| **Cell Death, % (IQR)** | 0.0 (0.0) | 2.3 (0.0 - 4.5) | 2.8 (0.9 - 5.8)* | 8.0 (5.6 - 10.3) | 0.9 (0.0 - 1.8) | 0.0019 |
| **Vacuoles, % (IQR)** | 0.0 (0.3 - 0.9) | 12 (6.5 - 17) | 3.7 (2.2 - 6.5) | 11.4 (10.5 - 18.3) | 2.6 (1.3 - 3.9) | 0.279 |

Blood smears from VEXAS patients (Met41Leu n = 2, Met41Thr n = 6, Met41Val n = 2, p.Gly40 Lys43del (c.118-1G>C) n = 1, c.118-2A>G n = 1) and HD (n = 20) were stained using MGG staining as described in Materials and Methods. Data are expressed as medians and IQR. p calculated using the Kruskal-Wallis test. Dunn’s post hoc test: *p<0.05, **p<0.01. Abbreviations are as follows: N, neutrophils; M, monocytes; L, lymphocytes; HD, healthy donors.

**Supplementary Table S8.** Cytokines and chemokines levels in VEXAS patients according to mutations.

|  | **HD** | **Met41Leu** | **Met41Thr** | **Met41Val** | **splice mutations** | **p** |
| --- | --- | --- | --- | --- | --- | --- |
| **IL-1β, pg/mL (IQR)** | 0.3 (0.0 -0.9) | ND | 0.8 (0.5 -1.5) | 3.8 (2.1 -5.5) | ND | 0.0233 |
| **IL-18, pg/mL (IQR)** | 285 (147 -459) | 721 (616 -826) | 704 (609 -857)* | 1096 (1072 -1119) | 771 (692 -850) | 0.0004 |
| **IL-1α, pg/mL (IQR)** | 0.0 (0.0 -0.2) | 1.8 (0.0-3.6) | 0.1 (0.0 -2.3) | 5.2 (2.1-8.2) | 2.6 (1.3 -3.8) | 0.0299 |
| **TNFα, pg/mL (IQR)** | ND | 1.0 (0.0 -2.0) | 0.0 (0.0-6.5) | 21.0 (17.0 -25.0) | 1.6 (1.0 -2.2) | 0.0003 |
| **IL-8, pg/mL (IQR)** | 0.4 (0.0 -1.1) | 3.6 (1.8-5.3) | 2.3 (1.5 -2.9) | 5.7 (4.8 -6.5) | 0.3 (0.0 -0.5) | 0.0158 |

Cytokines and chemokines levels in VEXAS patients (Met 41 Leu n = 2, Met 41 Thr n = 6, Met 41 Val n = 2, p.Gly40 Lys43del (c.118-1G > C) n = 1, c.118-2A > G n = 1) and HD (n = 20). Data are expressed as medians and IQR. p calculated using the Kruskal-Wallis test. Abbreviations are as follows: HD, healthy donors; ND, not detected.
